# Intra-orbital Malignant Melanoma: Role of Mr Imaging (a Case Report and Literature Review)

**DOI:** 10.5539/gjhs.v4n1p253

**Published:** 2012-01-01

**Authors:** Uduma Felix Uduma, Kamga Titalom

**Affiliations:** Department of Radiology Abia State University Teaching Hospital Aba, Nigeria Tel: 234 803 745 0099 E-mail: felixuduma@yahoo.com; Department of Ophthalmology, Polyclinic, Bonanjo Douala, Cameroon

## Abstract

Magnetic resonance imaging is a non-invasive modern imaging tool that can definitely diagnose malignant melanoma despite its anatomic localisations. This is borne out of tumour paramagnetic melanin pigment content. Melanin is known to shorten T1 and T2 relaxation times of protons thereby exhibiting hyperintense T1W and hypointense T2W signals, hence conferring some histiological diagnosis. This is unlike Amelanotic melanoma, other intra-orbital tumours and tumours in general that show usual hypointense T1W and hyperintense T2W signals. However a few mimics of signal characteristics of malignant melanoma like sub-retinal serous collection exist. This therefore needs additional MRI sequences like fat suppression with Gado-pentetate Dimeglumine enhancement for differentiations.

## 1. Introduction

Malignant Melanoma is malignant tumours of melanocytic origin, commonly seen on skin and various mucous membranes (Pandey *et al*, 2007, p30). This neoplasm results from malignant transformation of normal melanocyte (Garandawa *et al*, 2007, volume 9, number 2). Melanocytes are the cells that produce the pigment melanin, the main substance responsible for pigmentation of the skin and non-cutaneous sites) (Garandawa *et al*, 2007, volume 9, Number 2). Malignant melanoma is the commonest intra-ocular malignant neoplasms in adults. (Pandey *et al*, 2007, p30).

Evaluation of this intra-orbital melanoma ranges from ophalthmoscopy, Duplex Doppler ultrasonography (USS), Computed tomography (CT) to Magnetic resonance imaging (MRI) (3) (Bond *et al*, 1991, pp 459-466). But Malignant melanoma (MM) is uniquely suited for evaluation by MRI due to paramagnetic effect of the melanin pigment content of the tumour (Bond *et al*, 1991, pp 459-466, Uozum *et al*, 1990, pp 143-1469). This melanin shortens both T1 and T2 relaxation times causing hyperintense T1W and hypointense T2W images, thereby eliciting neuro-imaging interest and providing histiological diagnosis of this neoplasm.(Song *et al*, 1990, pp76-9)

Mucosal MM like intra-orbital malignant melanoma belongs to the class of tumours that on light microscopy may be confused with other malignancies unless the intra-cytoplasmic pigment or the melanoma cytoplasmic antigen is sought (Garandawa *et al*, 2007, volume 9, number 2). Such seeking could be by immuno-histiopathology of biopsied tumour sample or pre-operative MRI imaging. Thus, necessitating this report to highlight the need to equip African hospitals with modern imaging tools and immunohistopathological equipments

## 2. Case Report

**Figure 1:**
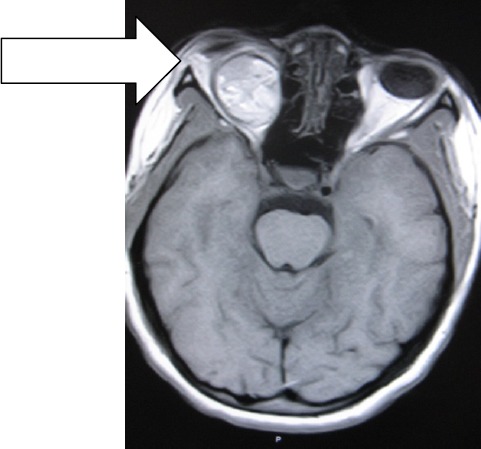
T1W

**Figure 2:**
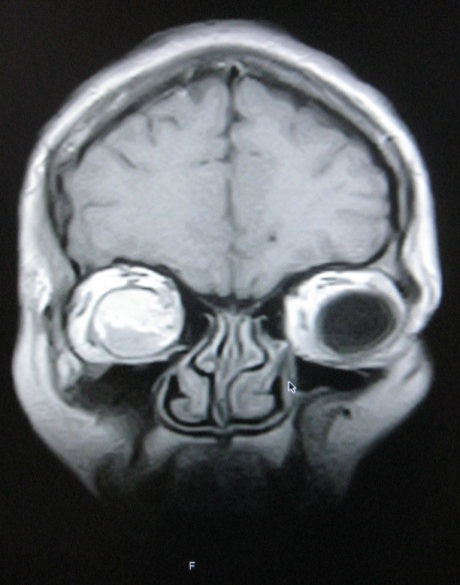
T1W

**Figure 3:**
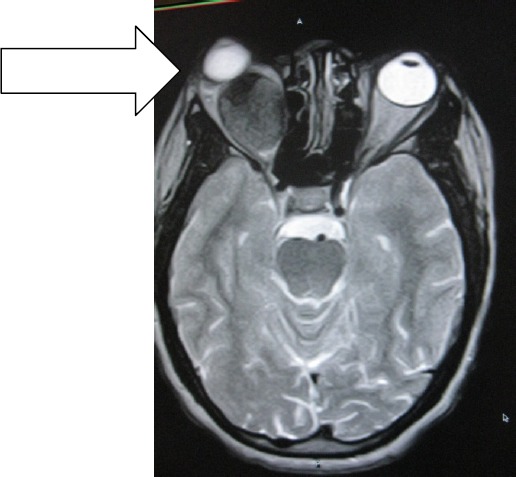
T2W

**Figure 4:**
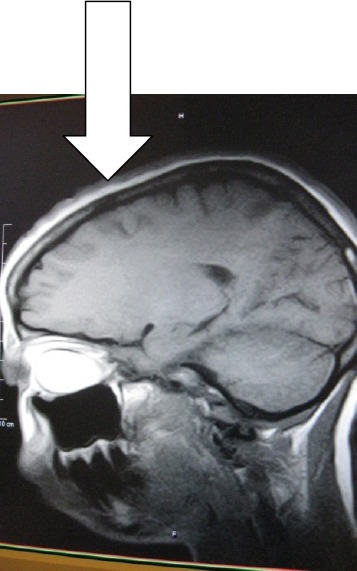
T2W

MB is an un-married 31year old Cameroonian Lady who was referred by the Ophthalmologist for brain MRI in Polyclinic Bonanjo, Douala. Her history was 7years of intermittent right eye pain with last one year progressive proptosis though without visual impairment. Brain MRI showed right intra-orbital, extra-ocular 52 X 33.3 X 20mm soft tissue well marginated mass that is hyperintense on T1W (Figure 1) and hypointense to vitreous on T2W (Figure 3) and FLAIR sequences reminiscent of paramagnetic melanin. This mass is markedly enhancing except an anterior-inferior part of the mass which is hypointense relative to the rest of the mass (Figure 2 coronal image). This suggests a recent intra-tumoral haemorrhage. Sagital T1W shows normal optic nerve with no optic foraminal nor intra-cranial extensions (Figure 4). There is proptosis of the right eye by a distance of 17.2mm (Figure 1). T1W intermediate and T2W hyperintense small polypoidal masses measuring 6.7mm, 6.4mm and 5.8mm in diameter are seen in the nasopharynx, right and left maxillary antra respectively. Ocular echography was not contributory, but shows near symmetrical globes with right and left measuring 23 ×19.4mm and 21.7 × 18.7mm in dimension. Patient visited the Opthalmologist with the result once, thereafter vanished denying us of any management and immune- histiology.

## 3. Discussion

Primary orbital melanoma is an exceedingly rare tumour that probably develops from congenital rests of neural crest cells in the orbit (Delaney *et al*, 2004, pp118-121). It represents less than 1% of primary orbital neoplasm and usually occurs in the presence of clinical or histiological evidence of ocular melanosis or blue nevus syndrom (Delaney *et al*, 2004, pp118-121). Naevi (mole) are aggregation of melanocytes that are present from birth, often do not make their appearance until puberty (2) (Gaeandawa *et al*, volume 9, number 2). During embryologic development, precursors melanocytes arise in the neural crest, as the fetus develops, these cells migrate to areas including the skin, meninges, the eye, upper oesophagus and the mucosal surface of the oral cavity, nasal cavity, paranasal sinuses, anorectal and urogenitalia (Garandawa *et al*, 2009, volume 9, number 2). Dark brown melanocytic pigmentation is normal observation in the conjunctiva, a condition referred as facial melanosis is especially evident bilaterally in more heavily pigmented races. (Goldberg *et al*, 1996, pp456-461) This condition is usually in the form of an excess production of melanin or hyperpigmentation by the melanocytes (forming an ephelis) or benign proliferation of melanocytes (forming a benign lentigo). (Goldberg *et al*, 1996, pp456-461)

Several factors are important in producing malignant transformation of the melanocyte, eg age, hormonal status, genetic predispositions, environmental factors, other risk factors eg trauma, dysplastic naevus syndrome, xeroderma pigmentosum, family history of melanoma, exposure to certain carcinogens and sunlight. For instance, Persons born in the Southern US had a relative risk of orbital and intra-orbital MM of 2.7 as compared with those born in the North (Turkar, 1985, pp 789-792). In general MM are more common in Caucasians than in Asia and Blacks population since lightly pigmented individual are at the higher risk of development of melanomas than are darkly pigmented individual (Garandawa *et al*, 2009, volume 9, number 2, Scott *et al*, 1976, pp 489-491, Lohman & Bridges, 1990, p 115). For example, uveal melanoma occurs 6-7 cases per million among Caucasians, 4.3 cases per million in US, most of which occurs in the white population and 0.25 per million in Japan (Kato *et al*, 2006, 404-9). Minutes areas of pigmentation that could predispose to the development of melanoma like small foci of pigmentation, subdermal or orbital melanocytosis are sometimes missed (Delaney *et al*, 2004, 118-121).

Primary orbital melanoma is a rare condition that is histopathologically similar to uveal melanoma). The commonest orbital malignant melanoma (MM) is uveal melanoma. This is because the uvea is the most vascularised portion of the eye, hence a substrate for primary and metastatic neoplasm (Peyman & Mafee, 1987, 471-88). Uveal melanoma is sub-classified into anterior uveal melanomas when the tumour arises from the iris and posterior melanomas when it arises from the choroid or ciliary body. The choroidal sub-type is the commonest MM among adults. WHO and International agency for Research on Cancer (IARC) reported 0.1-2.3 per 100,000 world-wide (Lutz *et al*, 1999, 1190-1193).

Women develop MM slightly more than men (Garandawa *et al*, 2007, volume 9, number 2). It is extremely rare for melanoma to occur before puberty, and the median age for diagnosis is in the late fortie (2) (Garandawa *et al*, 2007, volume 9, number 2). MM of mucosal surfaces eg head and neck are very rare according to American College of Surgeons Commission report (1998), 91% of all MM were cutaneous, only 1.3% were mucosal (Garandawa *et al*, 2007, volume 9, number 2, Chang *et al*, 1998, 1664-1678). Mucosal MM tends to occur in an older age group than the cutaneous counterpart from 5^th^ -8^th^ decade. Men are more affected than women in mucosal melanoma unlike in cutaneous melanoma (Garandawa *et al*, 2007, volume 9, number 2)

The aetiology of the paramagnetic relative enhancement seen in MM on proton MRI has been the subject of many investigations and has previously been ascribed to iron from associated haemorrhage or chelated metal ions, rather than directly due to melanin (Atlas *et al*, 1990, 547-554). Atlas *et al* indicated that T1 shortening correlates with increasing melanin content and not with increasing iron deposition, electron paramagnetic resonance (EPR) EPR-active metallic cation, necrosis or water content. Atlas *et al*, 1990, 547-554). In fact, they even found a probably unrelated statistical correlation between increased iron and T1 prolongation (Atlas *et al*, 1990, 547-554). Also T2 relaxation times did not correlate with the presence of any single factor other than proton density) Atlas *et al*, 1990, pp 547-554). Although the unique relaxation behaviour of non-haemorrhagic MM in vivo cannot be traced to a single cause, their data suggested that contrary to previous investigations, its strongly influenced by the presence of melanin rather than iron or other naturally occurring paramagnetic ions (Atlas *et al*, 1990, pp 547-554). The intensity of the tumour T1W signal but not the contrast enhancement is statistically associated with the degree of pigmentation of the tumour (Scot *et al*, 1998, pp897-899). Majority of MM with anticipated MR imaging melanotic pattern of high signal intensity relative to that of cortex on T1W and low signal intensity relative to that of cortex on T2W have more than 10% melanin containing cells (Isiakler *et al*, 1995, pp503-512). (Giovanni *et al*, 2006, pp605-8) Giovani *et al* also found a direct correlation between melanin content and T1W hyperintensity but no correlation between T2*W intensity and melanin (Giovanni *et al*, 2006, pp605-8).

Newer diagnostic modalities like MRI have modified the treatment of MM. Currently, Opthalmologists aim to save the eye and preserve any possible useful vision (Shanmugan *et al*, 1997, pp143-161). Supplementary modalities like MRI is warranted since dense vitreous may prevent view of fundus with opthalmoscopy. Also innumerable benign and malignant lesions may mimic the opthalmoscopic features of MM like choroidal haemorrhages (Shanmugan *et al*, 1997, pp143-161). Supplementary modalities are Fundus Fluorescein Angiography (FFA), Colour Doppler ultasonography (USS), Computed tomography (CT), Magnetic resonance imaging (MRI), Single photon emission tomography (SPECT) and Positron emission tomography (PET). Though FFA can differentiate certain Pseudo-melanomas from MM but Small choroid melanomas may not produce any appreciable change in angiogram due to absence of retinal pigment epithelium alteration and associated exudative retinal detachment (Shanmugan *et al*, 1997, pp143-161). ^123^I-IMP Sctingraphy using planar imaging or SPECT can provide specific localization of melanoma but the sensitivity of ^18^F-FDG-PET for diagnosis of MM is low due to high incidence of false negative results(13) (Kate *et al*, 2006,1404-9). CT is used when MRI is contraindicated and Non-contrast CT can only diagnosed 34% of uveal melanoma but increased to 75% in contrast CT (Kate *et al*, 2006, 1404-9). But CT might miss out brain metastasis from orbital melanoma < 2cm in diameter and the role of CT has been limited by poor tissue definition (Shanmugan *et al*, 1997, pp143-161, Golleri *et al*, 1991, pp27-34). Nearly all MM are confirmed by immunohistiological analysis using S-100 protein, HMB-45, Vimentin & Cytokeratin regardless of the site (Garandawa *et al*, 2009, volume 9, number 2)

MRI appears to be the most helpful and non-invasive imaging study of choice for evaluation of selected orbital lesions. MRI has been proven to be more sensitive and specific than USS in the detection of extra-ocular extension of uveal melanoma (Scott *et al*, 1998, pp 897-899). Information obtained from MR studies allow the identification of compounds such as melanin, met-haemoglobin, deoxyhaemoglobin and proteinacous fluid (Shanmugan *et al*, 1997, pp143-161). This is important because MM is a very vascular tumour, therefore the above blood degradation products, water and fat gives variations occasionally to the classical MR features of MM.

The MRI characteristics of orbital melanoma have been mainly attributable to paramagnetic properties of melanin. This melanin shortens T1 and T2 relaxation times leading to T1W hyperintense orbital melanoma which is a hypointense on T2W with respect to the hyperintense vitreous (Uozum *et al*, 1994, pp 76-9; Shanmugan *et al*,1997, pp143-161; Peyster *et al*, 1988, pp 773-9; Mafee *et al*,1989, pp 773-80) (Peyster *et al*, 1988, pp 773-9) Peyster *et al* reported these characteristic pattern in 93% of melanoma in their evaluations of intra-ocular tumours (De Potter *et al*, 1994, pp 340-8). De Potter *et al* also reported 95% of same patterns. However the presence of tumour necrosis containing water, presence of blood degradation products and iron content may explain the varying combinations of signal intensities such as decreased signal on T1W and/or increased signal on T2W (Shanmugan *et al*, 1997, pp143-161). He uses of fat suppression techniques help to improve the conspicuousness of the tumour and in differentiations from pseudo-melanomas and assess orbital extension (De Potter *et al*, 1994, pp 340-8). This technique combined with enhanced Gadopentetate Dimeglumine MRI images help to detect small intra-ocular mass with thickness of >1.8mm (22, 27) (Shanmugan *et al*, 1997, pp143-161; De Potter *et al*, 1994, pp 340-8).

Amelanotic melanoma exists, behaving just like any other tumour with hypointense or isointense T1W and hyperintense/isointense T2W (Ogwa, 2003, pp548-551, Escott, 2001, pp 625-639). On T1W Gado-pentetate enhancement, MM show as moderate to marked enhancement and larger tumours show more heterogenous enhancement than smaller tumours (Shanmugan *et al*, 1997, pp143-161). Preliminary reports based on limited number of cases have proposed that specific MR imaging patterns may permit a distinction between melanotic and amelanotic brain metastastasis in MM patients (Isiklar *et al*, 1995, pp 503-512). Rare orbital metastasis from cutaneous melanoma and contralateral choroidal melanoma have been reported (Sen Hadj Hamidi *et al*, 2009, pp416-420). The imaging features of metastatic melanomas are distinctive due to the presence of melanin and the propensity for haemorrhage. Both haemorrhage and melanin can produce T1W hyperintensity and T2W signal intensity loss (GIOV). T2W images improve detection of metastastic melanoma through T2W signal intensity loss due to susceptibity effect (Giovanni *et al*, 2006, pp 605-8)

The combination of T1W hyperintensity and T2W hypointensity signals have been seen in other intra-ocular lesions like serous retinal detachment, secondary sub-retinal fluid, uveal melanocytoma, choroidal osteoma, sub-acute choroidal/retinal haemorrhagic detachment, Retinoblastoma, Retinal capillary haemangioma, Focal retinal gliosis, Medulloepithelioma, and Inflammatory granuloma. This is due to proteinacious content or blood degradation product (Shanmugan *et al*, 1997, 143-161). Post-contrast T1W with fat suppression may help to differentiate these masses from MM as retinal detachment do not enhance and Choroidal haemangioma with high vascular flow and enhancement exhibit isointensity to slight hyperintensity on T1W and hyperintense T2 weighting which is isointense to vitreous.

## 4. Conclusion

MRI is pivotal in anatomical localisations and tumour extent of malignant melanoma. Interestingly, unlike in other tumours, it can suggest histiological diagnosis of Malignant melanoma based on unique signal intensities on different MRI sequences.
